# Supplementary material for: Flavonoids with Glutathione Antioxidant Synergy: Influence of Free Radicals Inflow
Source: Antioxidants (Basel). 2020 Aug 3;9(8):695. doi: 10.3390/antiox9080695 (PMC7465956; doi:10.3390/antiox9080695)
Supplement: Supplementary file 1 [file antioxidants-09-00695-s001.pdf]

antioxidants

MDPI

Supplementary material:

Flavonoids with Glutathione Antioxidant Synergy: Influence of Free Radicals Inflow

Igor Ilyasov, Vladimir Beloborodov, Daniil Antonov, Anna Dubrovskaya, Roman Terekhov, Anastasiya Zhevlakova, Asiya Saydasheva, Vladimir Evteev and Irina Selivanova

Table 1S. $ABTS^{\bullet+}$ self-bleaching at different initial absorbances

Initial $ABTS^{\bullet+}$ concentration, μM	Decrease rate, μM/min					
	0–1 min	1–10 min	10–15 min	15–20 min	20–25 min	25–30 min
33.3 (initial absorbance 0.50)	0.32±0.19	0.17±0.04	0.11±0.01	0.10±0.02	0.09±0.01	0.08±0.02
50.0 (initial absorbance 0.75)	0.28±0.11	0.20±0.05	0.12±0.04	0.11±0.01	0.09±0.01	0.08±0.02
66.7 (initial absorbance 1.00)	0.33±0.09	0.27±0.04	0.15±0.03	0.12±0.02	0.09±0.01	0.09±0.02
133.3 (initial absorbance 2.00)	0.51±0.10	0.28±0.03	0.15±0.05	0.13±0.02	0.09±0.02	0.08±0.03

Table 2S. Influence of different initial $ABTS^{\bullet+}$ concentrations on the antioxidant mixture effect of quercetin–glutathione 1:5 in the decolorization assay

Quercetin–glutathione 1:5				
$C_0(ABTS^{\bullet+})$, μM	Traditionally calculated mixture effect, %		Webb's simulation mixture effect, %	
133.00	-0.07±5.68	a	6.7±6.3	a
66.67	-7.15±3.20	d	4.8±0.3	b
50.00	-10.28±2.16	b	10.2±2.8	c
33.33	-6.26±6.47	a	8.7±5.9	a

a—statistically insignificant, $p > 0.05$; b, c, d—statistically significant with p-values less than 0.05, 0.01 and 0.001, respectively.

Table 3S. Influence of different component addition orders on the antioxidant mixture effect of the quercetin–glutathione 1:5 combination in the decolorization assay

Quercetin-glutathione 1:5								
Addition order	Traditionally calculated mixture effect, %				Webb's simulation mixture effect, %			
$ABTS^{\bullet+}$ + quercetin + (after 1 min) glutathione	-8.73	±	3.07	C	6.97	±	3.60	c
$ABTS^{\bullet+}$ + glutathione + (after 1 min) quercetin	-7.88	±	2.97	C	7.97	±	3.48	c

c – statistically significant with p-value less than 0.01

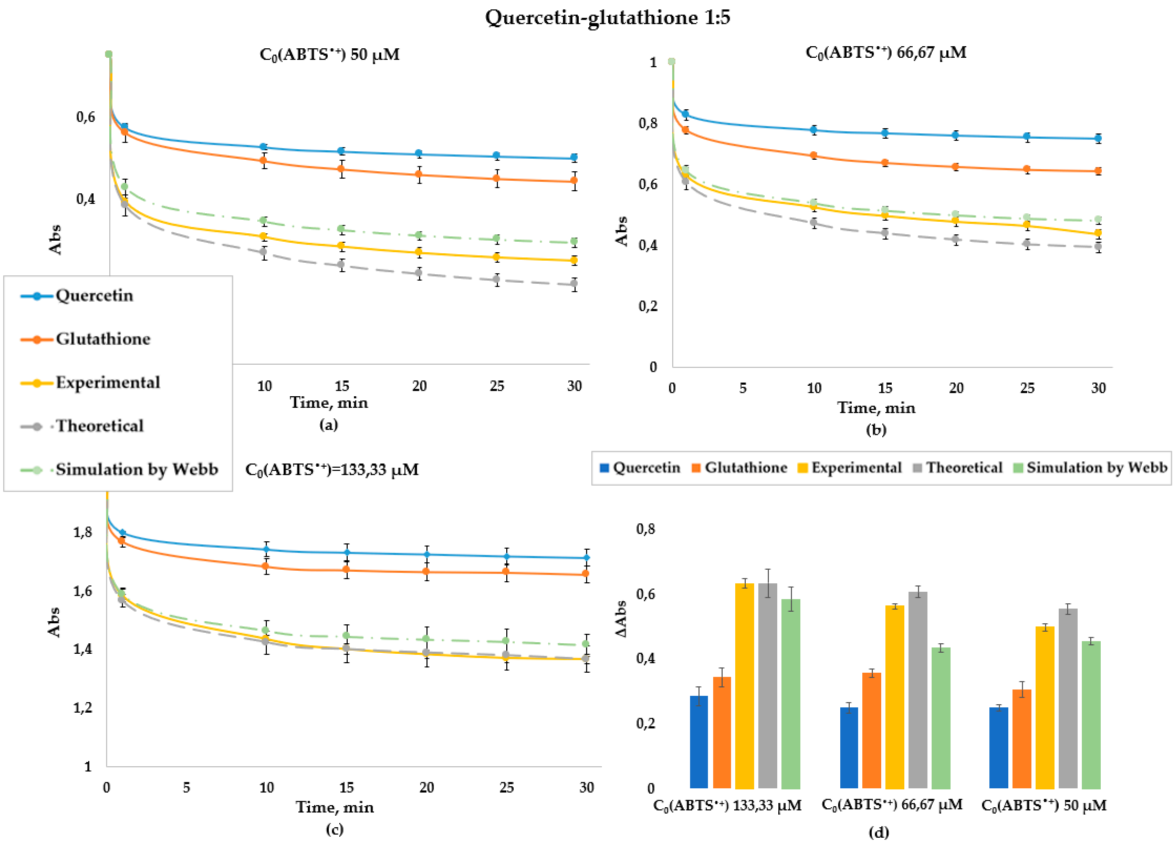

Figure 1S. Susceptibility to different initial ABTS•+ concentrations of the antioxidant mixture effect in the decolorization assay: 133.3, 66.7 and 50 µM, respectively; λ 730 nm, pathlength 1 cm. The ABTS•+ absorbance–time curves (**a**, **b**, and **c**) and absorbance loss (ΔAbs) diagram (**d**) demonstrates the experimentally derived results for individual components of combinations (quercetin and glutathione), the combination of quercetin–glutathione 1:5 (experimental), and theoretically calculated effect for combination obtained by simple summation (theoretical) or by applying Webb's simulation (simulation by Webb).

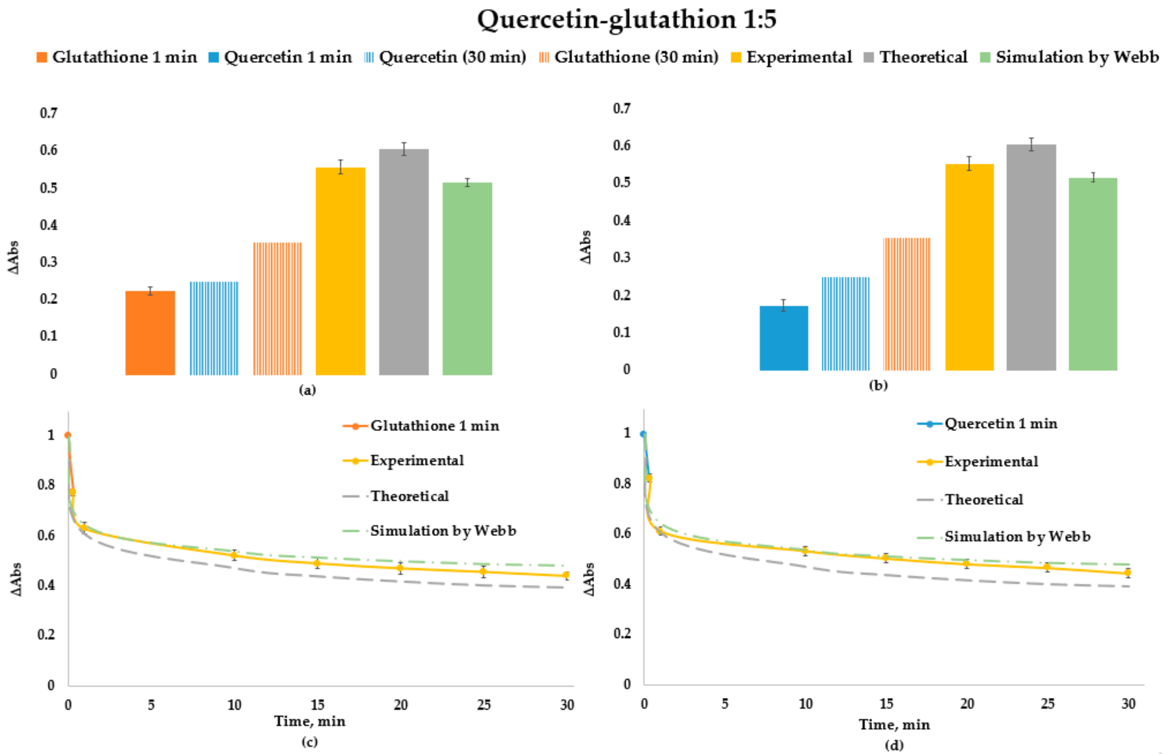

Figure 2S. Susceptibility to different component addition orders of the antioxidant mixture effect in the decolorization assay; λ 730 nm, pathlength 1 cm.
On the left (**a** and **c**): glutathione was mixed with ABTS$^{•+}$ and then quercetin was added 1 min later.
On the right (**b** and **d**): quercetin was mixed with ABTS$^{•+}$ and then glutathione was added 1 min later.
The ABTS$^{•+}$ absorbance loss (ΔAbs) diagrams (**a, b**) and absorbance–time curves (**c, d**) demonstrate the experimentally derived results for individual components: glutathione after 1 min incubation (glutathione 1 min), quercetin after 1 min incubation (quercetin 1 min), and final results for quercetin, glutathione (after 30 min) and quercetin–glutathione 1:5 (experimental, after 30 min), and theoretically calculated effect for the combination obtained by simple summation (theoretical) or by applying Webb's simulation (simulation by Webb).

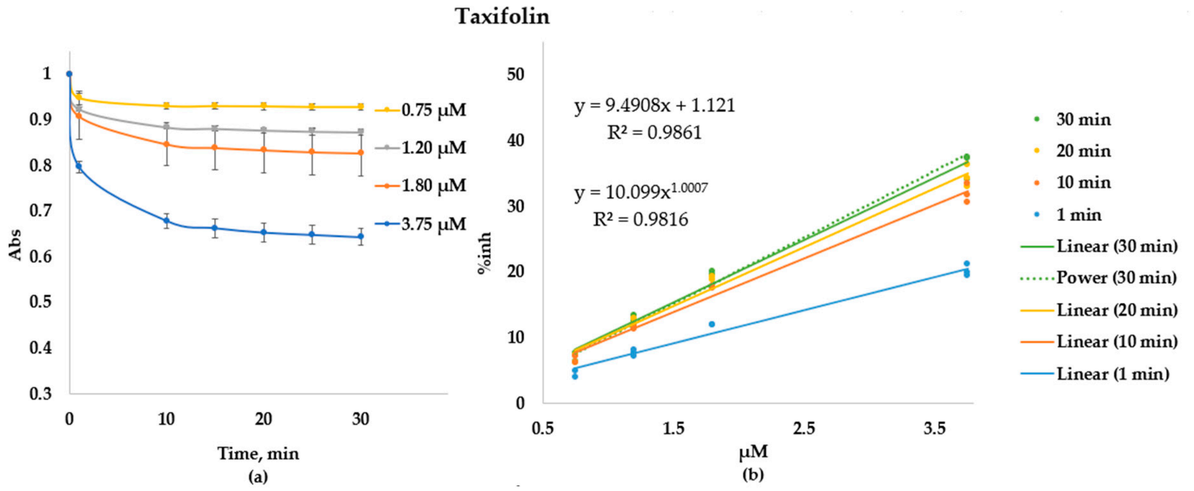
Taxifolin
Abs
Time, min
(a)
0.75 μM
1.20 μM
1.80 μM
3.75 μM
%inh
μM
(b)
y = 9.4908x + 1.121
R² = 0.9861
y = 10.099x^1.0007
R² = 0.9816
30 min
20 min
10 min
1 min
Linear (30 min)
Power (30 min)
Linear (20 min)
Linear (10 min)
Linear (1 min)

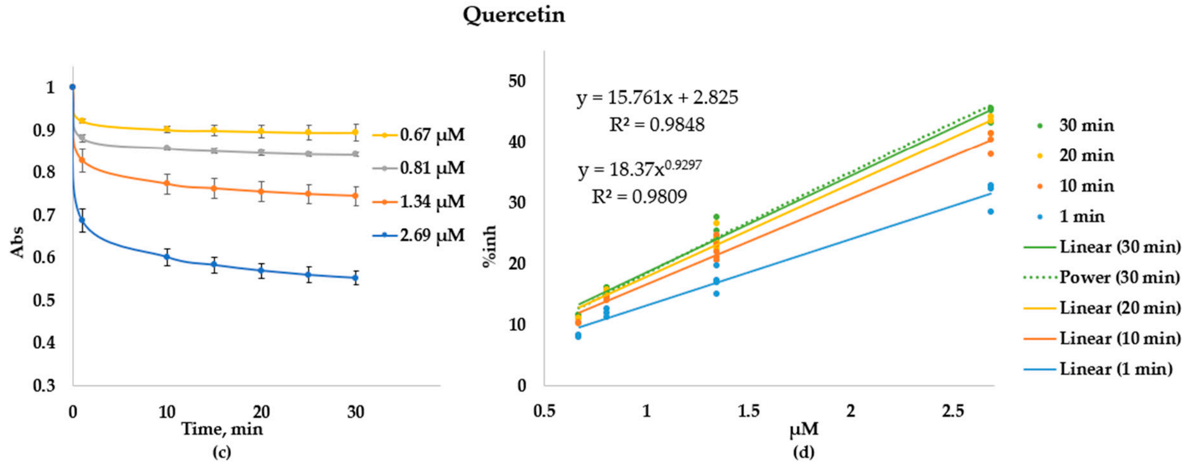
Quercetin
Abs
Time, min
(c)
0.67 μM
0.81 μM
1.34 μM
2.69 μM
%inh
μM
(d)
y = 15.761x + 2.825
R² = 0.9848
y = 18.37x^0.9297
R² = 0.9809
30 min
20 min
10 min
1 min
Linear (30 min)
Power (30 min)
Linear (20 min)
Linear (10 min)
Linear (1 min)

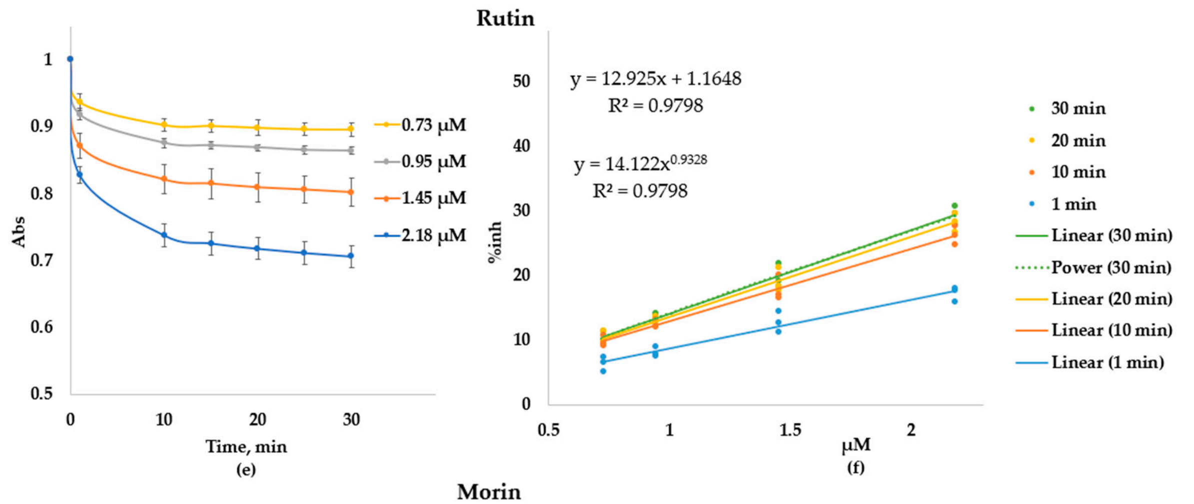

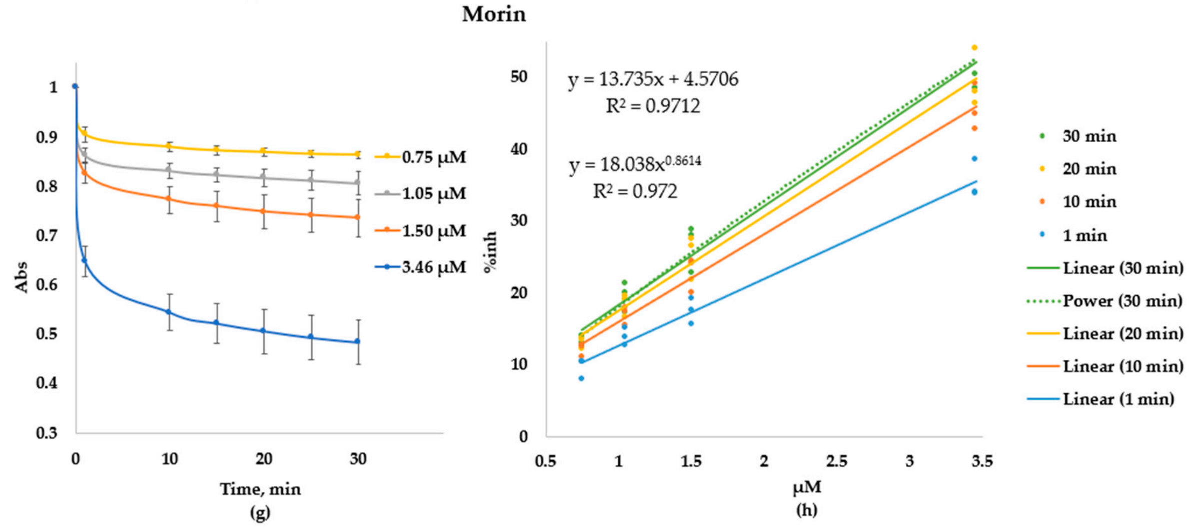

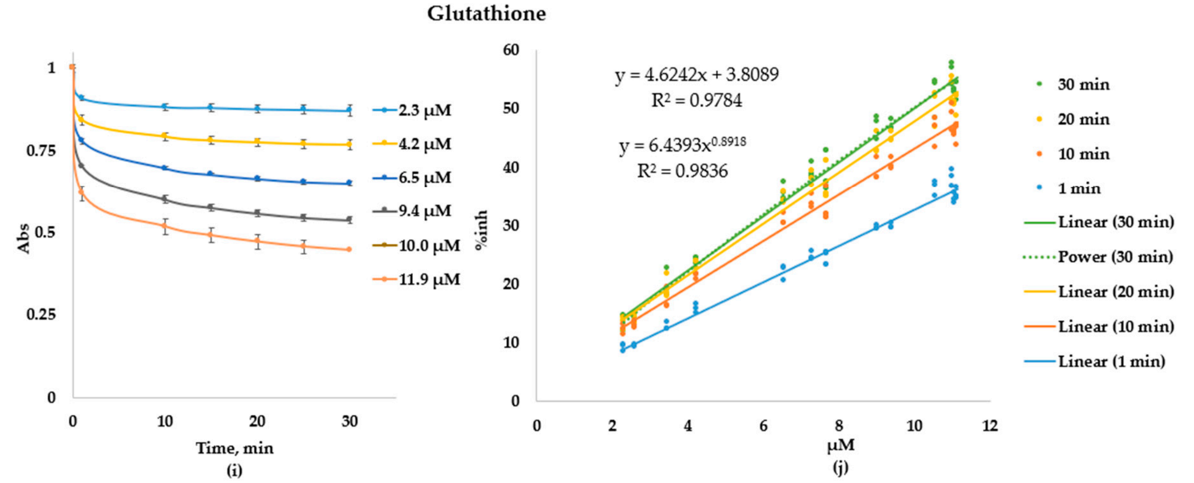

Figure 3S. The inhibition of $ABTS^{\bullet+}$ by flavonoids and glutathione.

On the left: absorbance decrease–time curves for taxifolin (**a**), quercetin (**c**), rutin (**e**), morin (**g**) and glutathione (**i**).

On the right: inhibition percentage–glutathione concentration regressions at different time points for taxifolin (**b**), quercetin (**d**), rutin (**f**), morin (**h**), and glutathione (**j**). λ 730 nm, pathlength 1 cm.

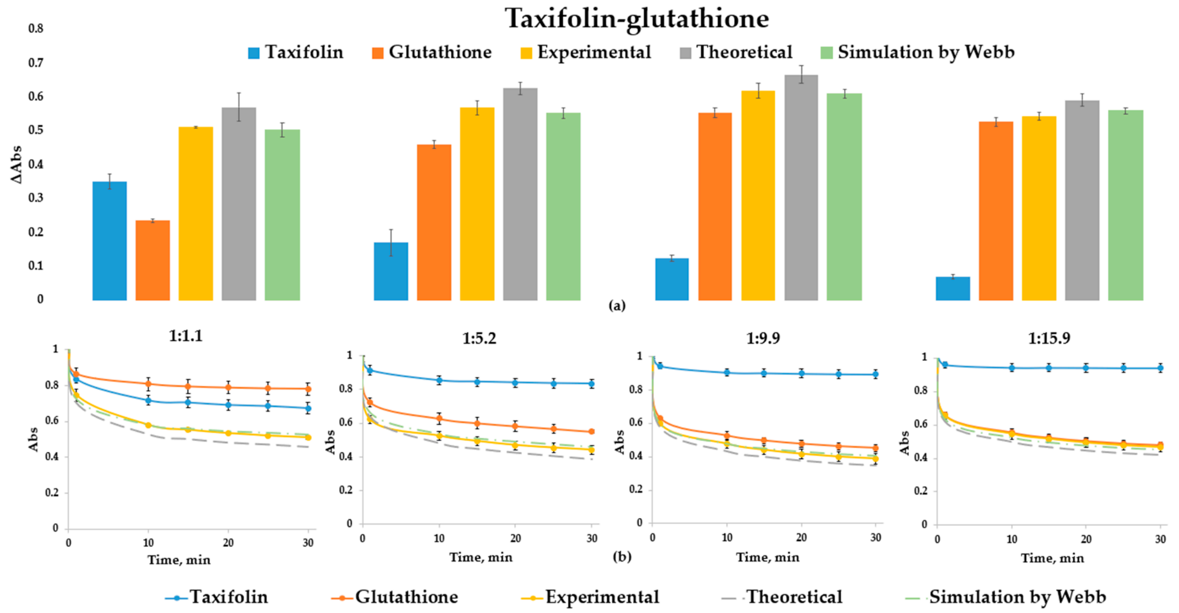
Taxifolin-glutathione
Taxifolin
Glutathione
Experimental
Theoretical
Simulation by Webb
ΔAbs
(a)
1:1.1
1:5.2
1:9.9
1:15.9
Abs
Time, min
(b)
Taxifolin
Glutathione
Experimental
Theoretical
Simulation by Webb

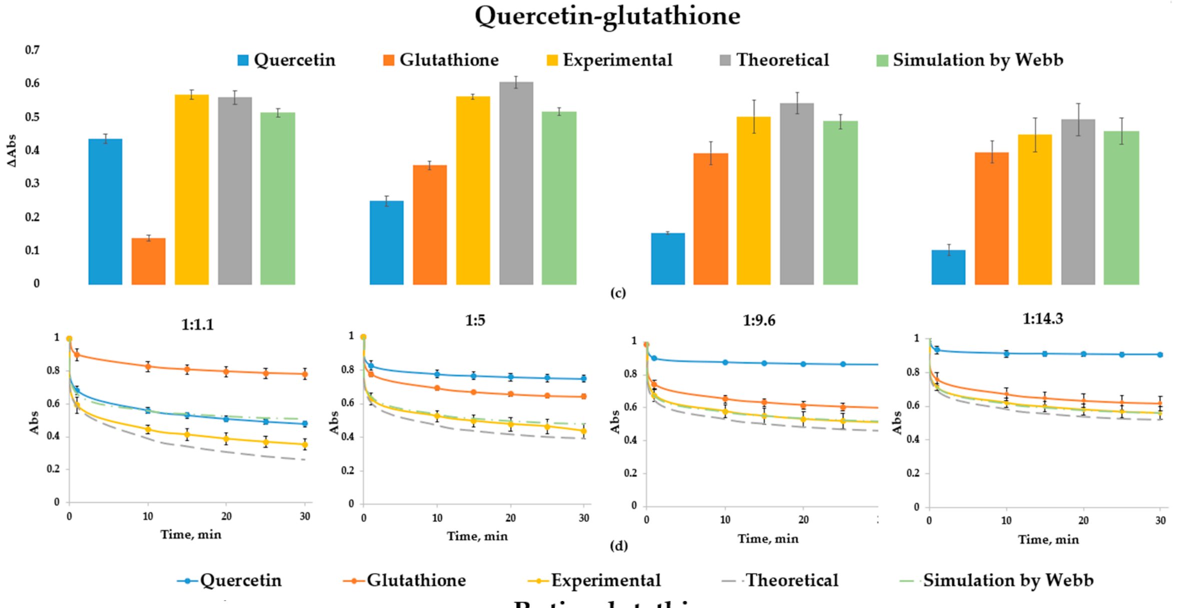
Quercetin-glutathione
Quercetin
Glutathione
Experimental
Theoretical
Simulation by Webb
ΔAbs
(c)
1:1.1
1:5
1:9.6
1:14.3
Abs
Time, min
(d)
Quercetin
Glutathione
Experimental
Theoretical
Simulation by Webb

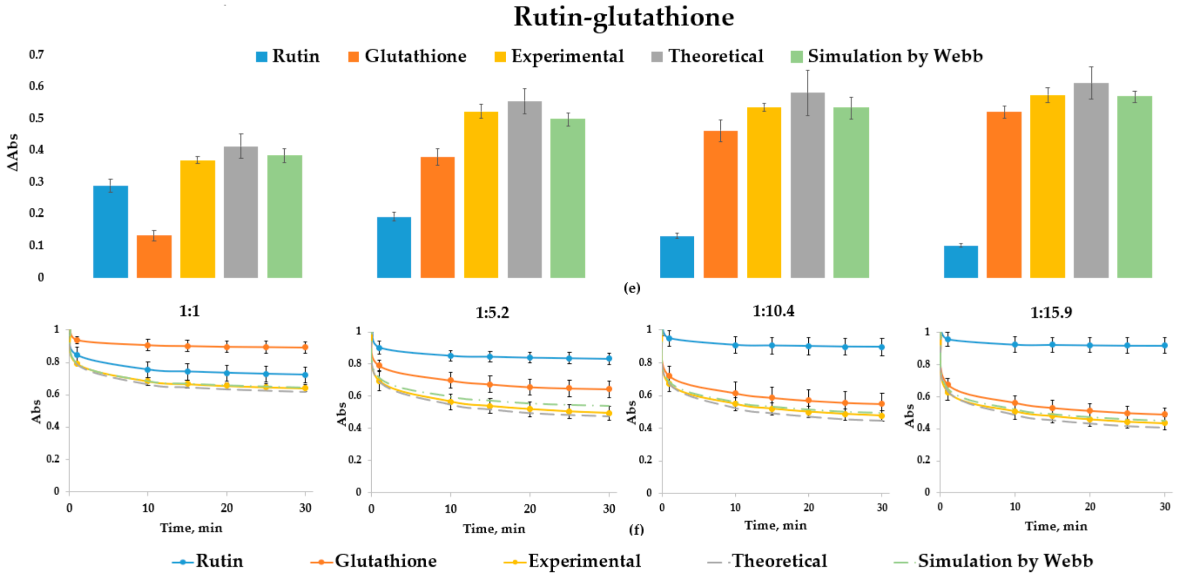
Rutin-glutathione
Rutin
Glutathione
Experimental
Theoretical
Simulation by Webb
ΔAbs
(e)
1:1
1:5.2
1:10.4
1:15.9
Abs
Time, min
(f)
Rutin
Glutathione
Experimental
Theoretical
Simulation by Webb

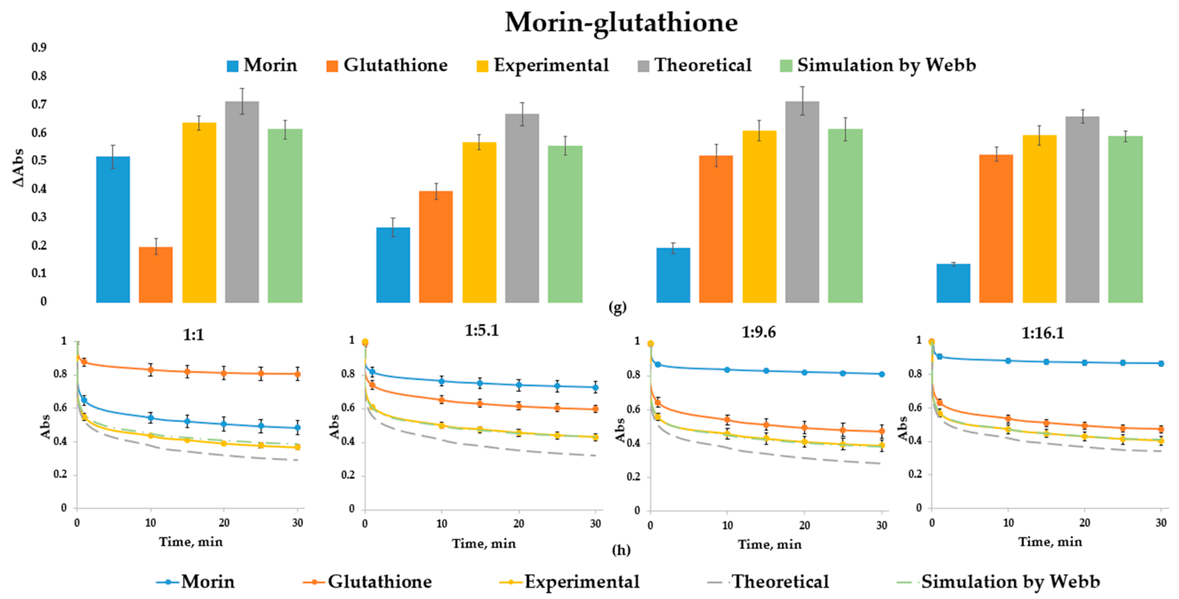

Figure 4S. Antioxidant mixture effect of flavonoid–glutathione combinations at different ratios in the decolorization assay: taxifolin–glutathione (**a**, **b**), quercetin–glutathione (**c**, **d**), rutin–glutathione (**e**, **f**) and morin–glutathione (**g**, **h**); λ 730 nm, pathlength 1 cm, incubation time 30 min .
The ABTS$^{•+}$ absorbance loss (ΔAbs) diagrams (**a**, **c**, **e**, **g**) and absorbance–time curves (**b**, **d**, **f**, **h**) demonstrate the experimentally derived results for individual components (flavonoid and glutathione), flavonoid–glutathione (experimental) and the theoretically calculated effect obtained by simple summation (theoretical) or by applying Webb's simulation (simulation by Webb).

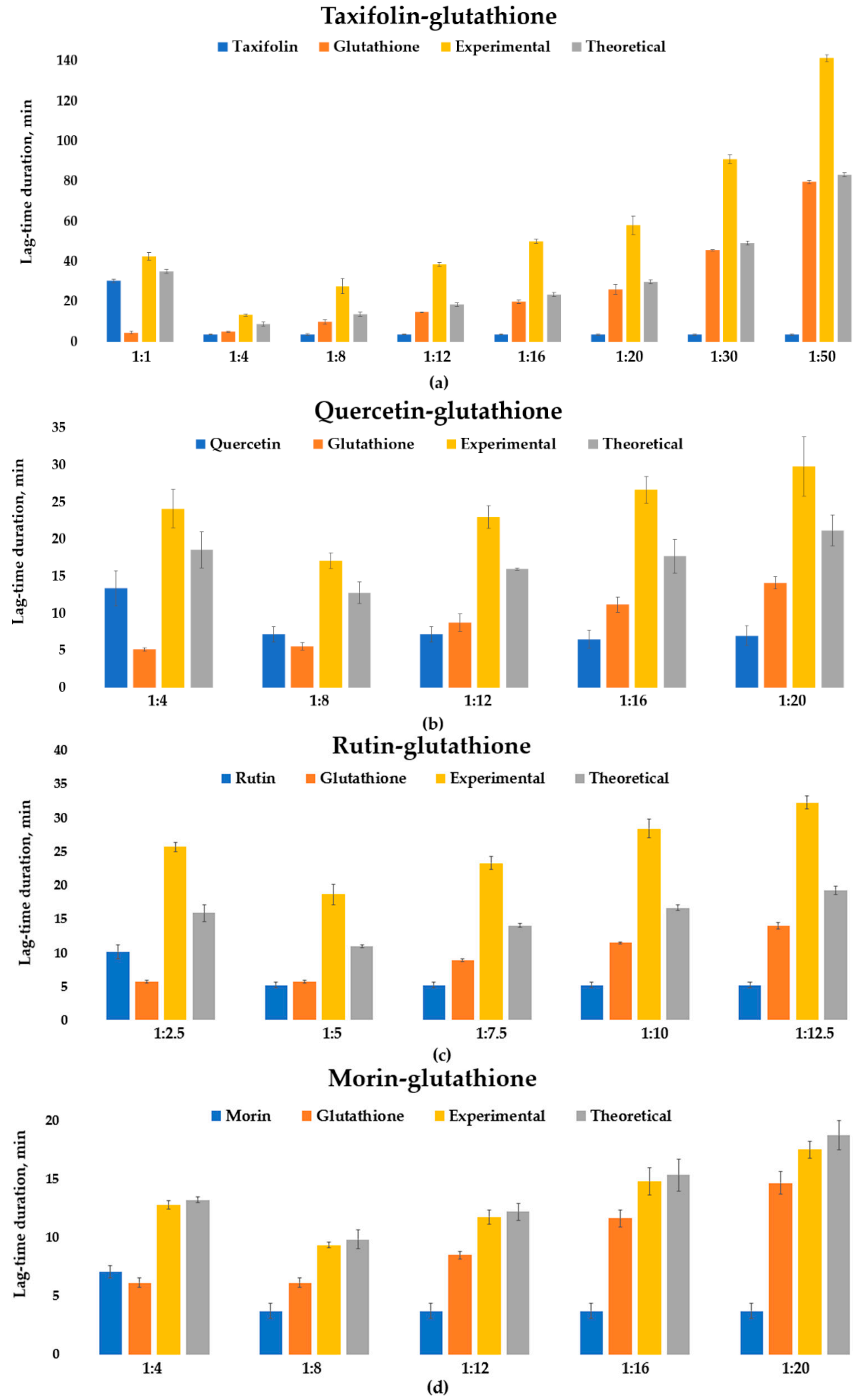

Figure 5S. Antioxidant mixture effect of flavonoid–glutathione combinations at different ratios in the lag-time assay: taxifolin–glutathione (**a**), quercetin–glutathione (**b**), rutin–glutathione (**c**) and morin–glutathione (**d**); λ 730 nm, pathlength 1 cm.

The lag-time diagrams (**a**-**d**) demonstrate the experimentally derived results for individual components (flavonoid and glutathione), flavonoid-glutathione (experimental) and the theoretically calculated effect obtained by simple summation (theoretical).

© 2020 by the authors. Licensee MDPI, Basel, Switzerland. This article is an open access article distributed under the terms and conditions of the Creative Commons Attribution (CC BY) license (http://creativecommons.org/licenses/by/4.0/).
